# A novel role of HLA class I in the pathology of medulloblastoma

**DOI:** 10.1186/1479-5876-7-59

**Published:** 2009-07-12

**Authors:** Courtney Smith, Mariarita Santi, Bhargavi Rajan, Elisabeth J Rushing, Mi Rim Choi, Brian R Rood, Robert Cornelison, Tobey J MacDonald, Stanislav Vukmanovic

**Affiliations:** 1 Center for Cancer and Immunology Research, Children's Research Institute, Children's National Medical Center, 111 Michigan Avenue NW, Washington, DC, USA; 2 Department of Pathology, Children's National Medical Center, 111 Michigan Avenue NW, Washington, DC, USA; 3 Department of Pediatrics, George Washington University School of Medicine, Washington, DC, USA; 4 Department of Neuropathology, Armed Forces Institute of Pathology, Washington, DC, USA; 5 Cancer Genetics Branch, National Human Genome Research Institute, NIH, Bethesda, Maryland, USA

## Abstract

**Background:**

MHC class I expression by cancer cells enables specific antigen recognition by the immune system and protection of the host. However, in some cancer types MHC class I expression is associated with an unfavorable outcome. We explored the basis of MHC class I association with unfavorable prognostic marker expression in the case of medulloblastoma.

**Methods:**

We investigated expression of four essential components of MHC class I (heavy chain, β2m, TAP1 and TAP2) in 10 medulloblastoma mRNA samples, a tissue microarray containing 139 medulloblastoma tissues and 3 medulloblastoma cell lines. Further, in medulloblastoma cell lines we evaluated the effects of HLA class I engagement on activation of ERK1/2 and migration in vitro.

**Results:**

The majority of specimens displayed undetectable or low levels of the heavy chains. Medulloblastomas expressing high levels of HLA class I displayed significantly higher levels of anaplasia and c-myc expression, markers of poor prognosis. Binding of β2m or a specific antibody to open forms of HLA class I promoted phosphorylation of ERK1/2 in medulloblastoma cell line with high levels, but not in the cell line with low levels of HLA heavy chain. This treatment also promoted ERK1/2 activation dependent migration of medulloblastoma cells.

**Conclusion:**

MHC class I expression in medulloblastoma is associated with anaplasia and c-myc expression, markers of poor prognosis. Peptide- and/or β2m-free forms of MHC class I may contribute to a more malignant phenotype of medulloblastoma by modulating activation of signaling molecules such as ERK1/2 that stimulates cell mobility.

## Introduction

The host immune system can be harnessed for the treatment of tumors because of the ability of T lymphocytes to specifically recognize tumor-associated antigens. CD8^+ ^T cells destroy tumor cells by perforin-dependent cytotoxic action, following recognition of MHC class I (HLA in humans) molecules at the cell surface [1]. Structurally, the MHC class I molecule is comprised of a 44-kD heavy chain, β2-microglobulin (β2m), and a peptide of 8–10 amino acid residues [2-4]. Presentation of antigenic peptides by MHC class I requires processing involving proteasome-mediated peptide generation from cytosolic proteins, peptide transport into the ER mediated by transporter associated with antigen processing (TAP), and peptide assembly with the heavy chain/β2m heterodimer [3,4]. TAP is a member of the ATP-binding cassette (ABC)-family of transporters and consists of the TAP1 and TAP2 subunits [3,5,6]. Although additional molecules are involved in assembly of MHC class I molecules, the heavy chain, β2m, TAP1 and TAP2 are especially important for functional expression of MHC class I and peptide presentation as deletion of any one of these four molecules results in a profound loss of cell surface expression [3,4].

Loss of MHC class I by tumor cells is thought to represent evasion of tumors from recognition by tumor-specific CD8^+ ^T cells [7,8]. Consistent with this notion, loss of MHC class I in cancers such as small cell lung carcinoma, pancreatic carcinoma, cervical cancer, colon cancer and melanoma is a negative prognostic factor [9-12]. In a carefully designed study, HLA class I expression was shown to dramatically decrease in secondary, relative to the primary lesions of breast carcinoma [13]. In another example involving glioma, loss of HLA expression was observed preferentially along the periphery of tumor samples, suggesting that this represents a stealth strategy that facilitates more aggressive tumor expansion [14]. However, a completely opposite correlation was observed in non-small cell lung cancer, uveal melanoma and breast carcinoma, where increased survival occurred in MHC class I negative tumors [15-17]. Interestingly, both positive (HLA-loss) and negative (HLA-down modulation) associations with the outcome of colorectal cancer have been observed in the same study [18]. The reasons for this counter-intuitive effect of MHC class I expression remain elusive.

Besides intercellular interactions, MHC class I molecules have also been implicated in *cis *modification of signal transduction. Heavy chains dissociated from β2m and peptides (called open conformers), and not the fully assembled MHC class I molecules, associate with a number of cell surface receptors, resulting in modulation of their activation [19]. Interestingly, addition of β2m, which reduces the proportion of open conformers, appears to either reduce [20] or enhance [21-24] signal transduction in different experimental systems. Thus, the direction of signaling modulation by MHC class I open conformers most likely depends on the identity of the associated receptor and can be manipulated by exogenous addition of β2m. Increased serum levels of β2m were found in several types of cancer [25-32] and were established as a poor prognostic factor in bronchial carcinoma [26], Hodgkin's lymphoma [27], multiple myeloma [31], renal cell carcinoma [29] and prostate carcinoma [32]. At present, it is unclear whether the effects of β2m are mediated through HLA class I.

Medulloblastoma is the most common pediatric central nervous system malignancy accounting for 30% of all pediatric brain tumors, with the highest prevalence between the ages of three and eight years [33,34]. Medulloblastoma has the tendency to disseminate throughout the central nervous system early in the course of the disease. The levels of β2m mRNA are significantly higher in metastatic than in non-metastatic forms of medulloblastoma [35], suggesting a potential association of β2m and/or HLA class I with aggressive behavior and poor prognosis in this type of tumor, as well. Invasiveness in medulloblastoma is mediated by several receptor activation pathways, such as platelet derived growth factor receptor and epidermal growth factor receptor [36]. Common components of these, as well as some other signaling pathways are ERK1/2 and AKT kinases. We show in this manuscript that a small fraction of medulloblastomas express high levels of MHC class I and that markers of poor prognosis are represented disproportionately more in specimens with high MHC class I expression. Furthermore, HLA class I may be involved in signaling modification as exogenously added β2m binds to HLA class I and enhances activation of ERK1/2 that, in turn, affects the mobility of medulloblastoma cells.

## Methods

### Generation of Tissue Microarray

Medulloblastoma tissue microarrays were generated as previously described [37]. Formalin-fixed, paraffin embedded tissue blocks were obtained from both the Armed Forces Institute of Pathology and Children's National Medical Center. More than 95% of tissues were obtained for diagnostic purposes, hence patients underwent no prior therapy. A neuropathologist (MS) marked two representative tumor core regions within each tissue block. The cores measuring 0.6 mm in diameter and 3–4 mm in height were isolated. A de-identified microarray was generated using 4–8 μm sections cut from the core blocks including: 139 medulloblastomas (47 classic, 40 anaplastic, 25 desmoplastic and 27 with unclassified histology), 21 primitive neuroectodermal tumors, 10 small cell carcinomas, 5 atypical teratoid rhabdoid tumors, 3 oat cell lung carcinomas, one each of ependymoblastoma and lymphoma, and 20 tissues of brain metastases from tumors of various origin. IRB approval was obtained for the construction and analysis of these tissue microarrays and all other tumor specimens investigated.

### Cell Lines

DAOY, D283 and D556 medulloblastoma cell lines were maintained at 37°C in a humidified atmosphere containing 5% CO_2 _in RPMI media supplemented with 10% Fetal bovine serum, 2 mM L-glutamine, 1 mM 2-mercaptoethanol, 100 U/ml penicillin and 100 μg/ml streptomycin.

### Antibodies

Polyclonal rabbit anti-human β2m and monoclonal mouse anti-human CD45 (clones 2B11 + PD7/26) were purchased from DakoCytomation, Carpinteria, CA. The hybridoma secreting HC-10 antibody, specific for HLA heavy chain epitope revealed in the absence of β2m and peptide [38], was kindly provided by Dr. Pan Zheng (University of Michigan School of Medicine, Ann Arbor, MI). Anti- TAP1 (NOB-1) and TAP2 (NOB-2) antibodies [39] were provided by Dr. S. Ferrone (Hillman Cancer Center, University of Pittsburgh Cancer Institute, Pittsburgh, PA). The W6/32 monoclonal antibody is specific for HLA A, B and C and specifically to the combined epitope contributed by the α2 and α3 domains of the heavy chain and β2m [40-42]. mAb rabbit anti-human GAPDH, rabbit anti-human phospho-p44/p42 MAP kinase (Thr202/Tyr204), mAb rabbit anti-human p44/p42 MAP kinase, rabbit anti-human phospho-AKT (Ser473), rabbit anti-human AKT, mAb mouse anti-human phospho-EGF receptor (Tyr1068), rabbit anti-human EGF receptor, rabbit anti-human phospho-PDGFRβ (Tyr751) were purchased from Cell Signaling (Beverly, MA), anti-c-myc from Abcam (Cambridge, MA), rabbit anti-human PDGFR- β (Santa Cruz) and anti-rabbit IgG HRP-linked antibody (Cell Signaling). HC-10 and W6/32 antibodies were partially purified from hybridoma supernatants using the centriplus YM-100 (Millipore) regenerated cellulose filters that have a molecular weight cutoff of 100,000 Da. Antibody concentration after purification was 2 mg/ml.

### Immunohistochemistry (IHC)

Slides containing tissue arrays were deparaffinized and rehydrated. The primary Abs HC-10, TAP1 and TAP2 required an additional heat-induced epitope retrieval step (Target Retrieval Solution pH 9 or Target Retrieval Solution, pH6.1, DakoCytomation, Carpinteria, CA). Slides were blocked with biotin (Biotin Blocking System, DakoCytomation) and processed using the 3-step streptavidin-biotin-immunoperoxidase staining system (LSABTM2+ System-HRP, DakoCytomation, Carpinteria, CA). Diaminobenzidine (DAB) was the chromogenic substrate. Mayers hematoxylin was used as a counterstain and followed with an ammonia wash. Slides were mounted using an aqueous mounting medium (Faramount, DakoCytomation). Controls consisted of parallel sections without primary antibody. Stainings for heavy chain, β2m, TAP1, TAP2 and CD45 were assigned scores of 0–3 [43] based on the percentage of stained cells (0- no staining; 1- less than 10% cells stained; 2- 10–50% cells stained; 3- >50% cells positive). The staining for c-myc was not restricted to particular cells, but was rather present or absent throughout the tumor. Hence, c-myc scores were assigned 0 (absent staining), 1 (weak diffuse staining) or 2 (strong diffuse staining). Each score was an average of the two samples graded for each individual tumor in the array. Each slide was evaluated by three independent graders including two neuropathologists. The images were acquired using the following equipment: Microscope – Carl Zeiss Axioskop; Lenses- Achroplan 40× and Achroplan 20×; Camera – Carl Zeiss Axio Cam HRC; Acquisition software- Axio Vision Rel. 4.3.

### Flow Cytometry

Cells were incubated with either 5% FBS in PBS (controls) or W6/32 or HC-10 supernatant for 1 hour. After 3 rinses, cells were incubated with PE labeled donkey anti-mouse IgG (H+L) (eBiosciences, San Diego, CA) for 30 minutes at 4°C. Cells were rinsed 3 times and fixed in cytofix (BD Sciences, San Diego, CA).

### RT-PCR

Total cellular RNA, obtained from 10 frozen medulloblastoma specimens, was subjected to the SuperScript First-strand synthesis system for RT-PCR (Invitrogen, Carlsbad, CA) to generate the cDNA for the PCR reactions. RT-PCR were performed using 35 cycles of 30 seconds at 94°C, 1 minute at the annealing temperature, and 1 minute at 72°C (with the exception for HLA-A and HLA-B where the last cycle was for 1 minute 30 seconds at 72°C). The annealing temperatures were 57°C for TAP2 and β2m, 58°C for β-actin and CD45, 59°C for TAP1 and 60°C for HLA-A and HLA-B. The primer sequences were: 5'-GAGACATCTTGGAACTGGAC-3' and 5'-CTCTGAGTGAGAATCTGAGC-3' (forward and reverse, TAP1), 5'-GTACAACACCCGCCATCAG-3' and 5'-GGACGTAGGGTAAACGTCAGC-3' (TAP2), 5'-CTCGCGCTACTCTCTCTT-3' and 5'-AAGACCAGTCCTTGCTGA-3' (β2m), 5'-TATAGTCGACCACCCGGACTCAGAATCTCCT-3' and 5'-ATATGGATCCATCTCAGTCCCTCACAAGA-3' (HLA-A), 5'-TATAGTCGACCACCCGGACTCAGAGTCTCCT-3' and 5'-ATATGGATCCATCTCAGTCCCTCACAAGA-3' (HLA-B), 5'-ACCTGTACGCCAACACAGTG-3' and 5'-GCCATGCCAATCTCATCTT-3' (β-Actin), 5'-CTGAAGGAGACCATTGGTGA and 5'-GGTACTGGTACACAGTTCGA-3' (CD45). The reactions were loaded onto a 1% agarose gel and products were visualized by ethidium bromide staining.

### Western Blotting

DAOY and D283 cells were cultured in serum-free media for 24 hrs at 37°C. Cells were washed twice and treated with serum-free RPMI with or without human β2m (Lee Biosolutions, Inc, St. Louis, Missouri) or monoclonal antibodies for indicated times, at 37°C. Signaling was halted by the addition of ice cold PBS and cells were lysed with 1× cell lysis buffer (Cell Signaling, Danvers, Massachussetts) supplemented with PhosSTOP phosphatase inhibitor cocktail and complete mini protease inhibitor cocktail (Roche, Indianapolis, Indiana). Lysates were centrifuged at 14,000 rpm for 15 minutes to remove cell debris, boiled for 5 minutes in loading buffer and separated by a 4–12% Bis-Tris Gel (Invitrogen, Carlsbad, California). Membranes were blocked in 5% milk in TBST for 1 hour at room temperature. Primary and secondary antibodies were diluted in 3% BSA in TBST and incubated with the membrane at 4°C overnight and at room temperature for 1 hour, respectively. Signal was detected using the enhanced chemiluminescence system (Pierce, Rockford, IL). Densitometry quantification of the bands was performed using Quantity One software (Bio-Rad, Hercules, CA), according to manufacturer's instructions. The Band 1/Band 2 ratio was obtained by dividing differences between intensity units observed in the square areas containing specific bands and an identical blank area drawn in the immediate vicinity of the band, according to the following formula: Band 1/Band 2 ratio= (Band 1-blank)/(Band 2 -blank).

### Wound scratch assay

Wound scratch assay was used to evaluate tumor cell migration [44,45]. DAOY cells were plated in a 60 mm dish at 40% confluency and grown overnight. After 24 hours the cells were scraped down the middle of the plate with a 200 μl pipette tip to induce the resemblance of a wound and washed twice with serum-free media. Cells were cultured with serum free media with or without 2 μg/ml β2m for 24 hours. Images of the wound were taken at 0 and 24 hours. The area of the wound was calculated using the Axiovision system by tracing the cleared space. Area was measured in μM2. The ratio of 24 hours to 0 hours was calculated for each sample.

## Results

### Medulloblastoma expression of MHC class I antigen

**Figure 1:**
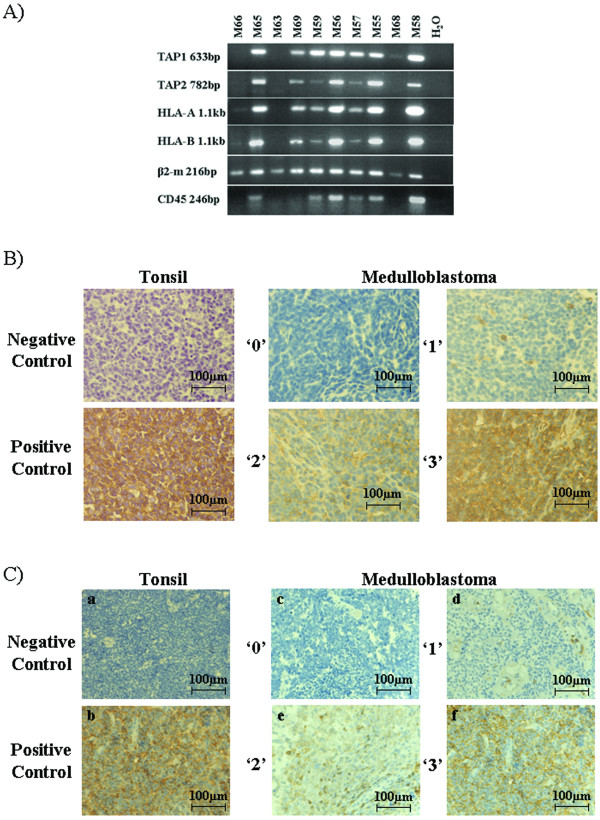
**Detection of classical MHC class I components in medulloblastoma samples**. A) RNA was isolated from frozen tissue sections of individual medulloblastoma patients. Indicated are the specificities of the primers used and the size of each amplification product. Negative control (H_2_O) contained no cDNA template. B) Representative sections showing staining with the heavy chain-specific monoclonal antibody HC-10 (×40 magnification). Tonsil tissue sections processed identically except for the absence (negative control) or presence (positive control) of primary HC-10 monoclonal antibody. Examples of medulloblastoma sections graded as 0, 1, 2 and 3. C) Representative sections showing staining with the CD45-specific monoclonal antibody (×40 magnification). Tonsil tissue sections processed identically except for the absence (a) or presence (b) of primary monoclonal antibody. Examples of medulloblastoma sections graded as 0, 1, 2 and 3.

Expression of MHC class I in medulloblastoma has been reported in two studies with conflicting results [46,47]. We therefore examined the MHC class I expression in our collection of medulloblastoma specimens. The expression of the essential components of the MHC class I antigen-processing machinery: HLA heavy chains (A and B), β2m, TAP1 and TAP2 was first evaluated using RNA from ten frozen medulloblastoma specimens. β2m was expressed in all samples, while heavy chains and TAP subunits were detected in seven samples (Fig. 1A). An essential element of this analysis was the assignment of MHC class I expression to the tumor cells and not to the leukocytes infiltrating the medulloblastoma tissues. CD45 detection was used to evaluate contamination by leukocyte-derived RNA. CD45 was undetectable in four samples. Three of the CD45-negative samples (M66, M63 and M68) expressed β2m and not the other three components, indicating that the majority of medulloblastomas fail to express MHC class I. The fourth sample (M69) expressed all four components.

Because of relatively frequent leukocyte infiltration, the ability to discriminate RNA from tumor versus stromal host cells was low in many medulloblastoma samples. IHC analysis of medulloblastoma tissues is advantageous in this respect, because areas of leukocyte infiltration and MHC class I expression can be directly visualized and compared to tumor cell expression in each individual sample. We therefore analyzed HLA heavy chains, β2m, TAP1, TAP2 and CD45 intracellular and/or cell surface expression using medulloblastoma tissue microarrays. Representative staining patterns for HLA heavy chains are shown in Fig. 1B. Of the 106 evaluable specimens 87% showed absent or faint heavy chain positivity (56% scored 0 and 31% scored 1), while scores 2 and 3 were observed in 5% and 8% of tissues, respectively (Table 1). To gain a better molecular understanding of MHC class I expression, medulloblastoma arrays were stained with antibodies to TAP subunits and β2m (Table 1). In agreement with the mRNA analysis, the majority of medulloblastoma tissues expressed high levels of β2m protein. Surprisingly, similarly high levels of TAP1 were seen, while staining of TAP2 was intermediate (Table 1). The 14 samples with scores 2 or 3 for staining with HC-10 antibody also displayed high levels of TAP1, TAP2 and β2m (except for TAP2 in two samples). Therefore, we conclude that there is a small subset of medulloblastomas that may express all components required for functional MHC class I molecules, whereas at least one essential component (heavy chain or TAP2) is missing in most tumor samples.

**Table 1:** Summary of HLA class I and CD45 staining scores for medulloblastoma array.

Antibody	Immunohistochemistry scores				Total
	0	1	2	3	

HC-10	59 (55.7%)	33 (31.1%)	5 (4.7%)	9 (8.5%)	106 (100%)

β_2_m	2 (1.6%)	21 (16.9%)	28 (22.6%)	73 (58.9%)	124 (100%)

TAP1	0 (0.0%)	11 (8.9%)	39 (31.5%)	74 (59.7%)	124 (100%)

TAP2	16 (13.1%)	64 (52.5%)	27 (22.1%)	15 (12.3%)	122 (100%)

CD45	60 (58.3%)	41 (39.8%)	1 (0.95%)	1 (0.95%)	103 (100%)

CD45*	6 (42.9%)	6 (42.9%)	1 (7.1%)	1 (7.1%)	14 (100%)

*CD45 scores on a subset of specimens with scores of 2 or 3 for all components required for MHC class I expression (HC-10, β_2_m, TAP1 and TAP2).

To determine whether expression of the components of MHC class I processing machinery could be ascribed to infiltrating leukocytes, we stained the medulloblastoma microarray with anti-CD45 antibody (Fig. 1C). Of the 103 evaluable medulloblastoma specimens, 58.3% were negative for infiltration with 39.8% receiving a score of 1 and 0.95% a score of 2 and 3 each (Table 1). The positive staining samples showed leukocytes present in the tumor, but few that had migrated away from the blood vessels into the tissue. The low levels of infiltration appear to be indicative of the expected poor immune response associated with medulloblastomas. Of the 14 MHC class I-high samples, 6 were negative for leukocyte infiltration and 6 received a score of 1. Scores of 2 and 3 were noted in one sample each. Therefore, contamination by infiltrating peripheral white blood cells cannot account for positive heavy chain, TAP1, TAP2 and β2m staining in most of these cases and MHC class I expression appears to be genuinely derived from medulloblastoma cells.

### Association of MHC class I expression and anaplastic medulloblastoma subtype

Anaplastic histopathology [48,49] and c-myc expression [49-51] are negative prognostic markers for medulloblastoma. To test whether MHC class I expression had any prognostic value for medulloblastomas, we evaluated the distribution of histological subtypes and expression of c-myc in specimens with scores 2 or higher for heavy chain versus those that scored less than 2. The reason for the cut-off at score 2 is that score 1 was given if only up to 10% cells in the specimen stained positive. In contrast to the diffuse character of the anaplasia and c-myc expression, we considered that the impact, if any, of so few positive cells on the overall histology of the tumor could not have been significant.

**Figure 2:**
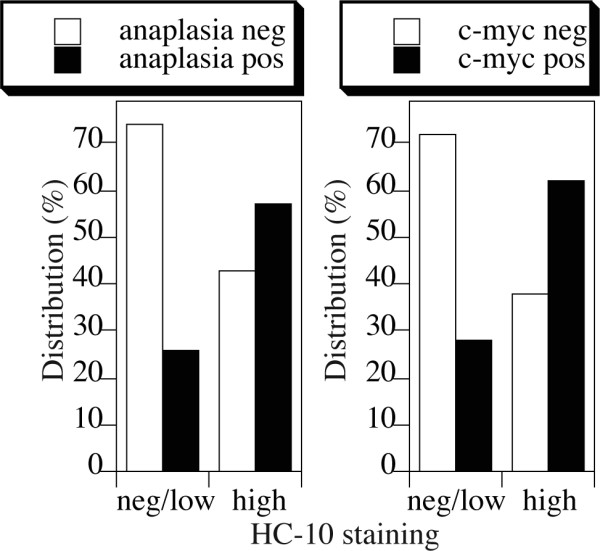
**Association of MHC class I expression with anaplastic histopathology and c-myc expression**. Distribution of the anaplasia or c-myc expression in the medulloblastoma array specimens exhibiting low/negative (HC-10^neg/low^) or high levels (HC-10^hi^) of HLA class I heavy chains. Fisher exact test showed statistically significant differences in distribution with p = 0.0251 for histopathology (n = 105) and p = 0.0257 for c-myc expression (n = 88).

Of the 106 samples evaluable for HC-10 staining, 31 were anaplastic, 26 classic, 23 desmoplastic and 26 histologically unclassified (without evidence of diffuse anaplasia). Of the fourteen MHC class I-high medulloblastomas 8 were anaplastic, 3 classic, 1 desmoplastic and 2 unclassified. Similar findings were observed when c-myc expression was considered. Of the 88 samples that were evaluable for both HC-10 and c-myc, 59 scored 0, 23 scored 1 and 6 scored 2 for c-myc expression. In contrast, in the MHC class I-high population there were 5 c-myc negative samples, and 6 and 2 with scores 1 or 2 respectively. The frequency of anaplastic versus any other histological subtype, or c-myc negative versus c-myc positive specimens in the MHC class I-high versus MHC class I-low or -negative medulloblastoma specimens is significantly different (Fig. 2), with p values being 0.0251 for histopathology (n = 105) and 0.0257 for c-myc expression (n = 88).

### Binding of exogenous β2m alters the balance of open and closed MHC class I conformers in medulloblastoma cell line

**Figure 3:**
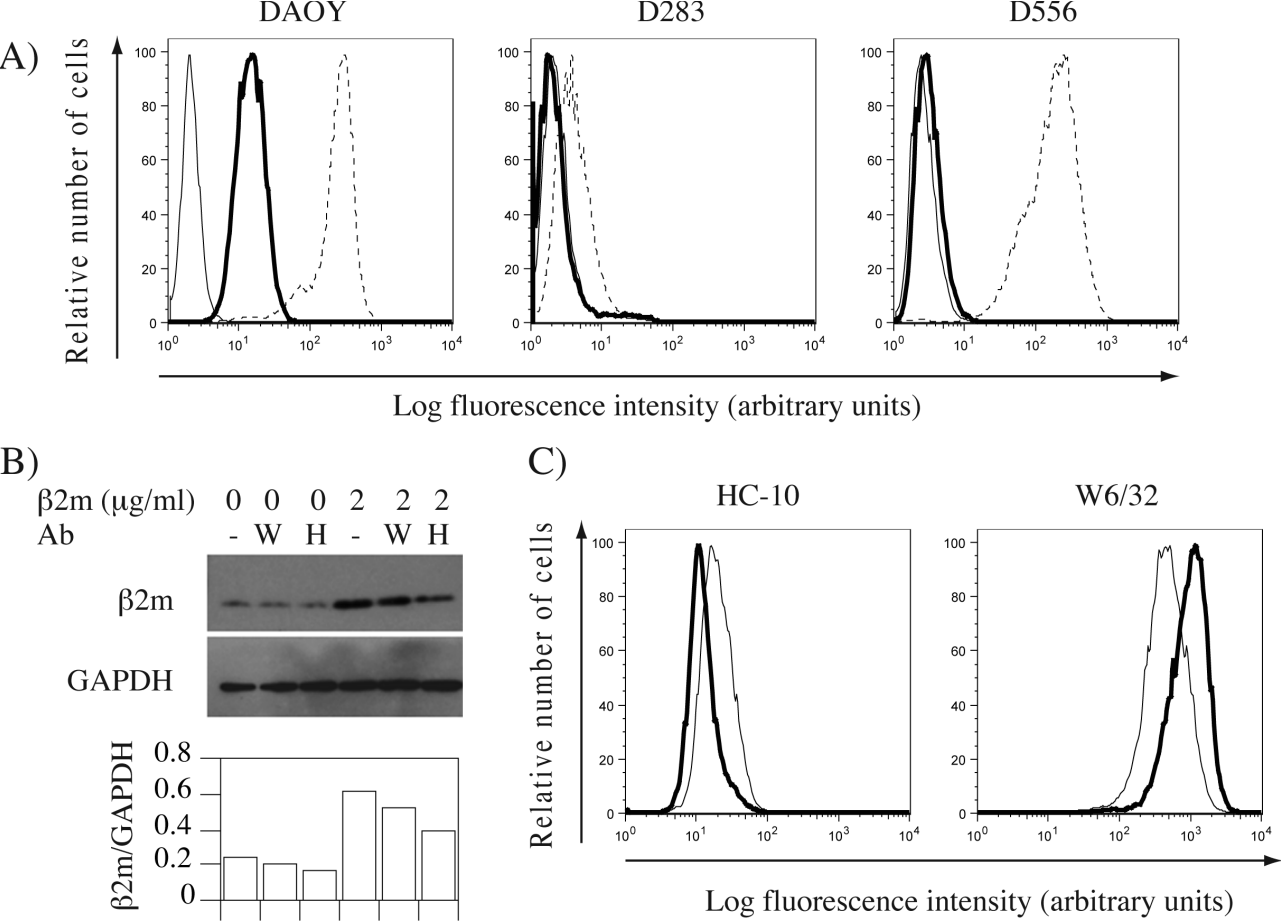
**Binding of exogenous β2m tips the balance of open and closed cell surface HLA class I forms**. A) Flow cytometry analysis of DAOY, D283 and D556 medulloblastoma cell lines stained with HC-10 (bold lines) or W6/32 (dashed lines) monoclonal antibody followed by PE-conjugated anti-mouse Ig, or control samples with primary antibody omitted (plain lines). B) Exogenous β2m in the absence or presence of W6/32 (W) or HC-10 (H) monoclonal antibodies was added to DAOY cells. Cell lysates were probed by β2m- or GAPDH-specific antibodies. Bar graph indicates quantitative ratios of β2m to GAPDH. C) The effect of exogenous β_2_m on levels of open and closed conformers in DAOY cells. Untreated (plain lines) or β_2_m-treated (bold lines) DAOY cells were stained with antibodies specific for open (HC-10; left) or closed (W6/32; right) MHC class I conformation followed by PE-conjugated anti-mouse Ig, and analyzed by flow cytometry.

The levels of open and closed conformer were respectively analyzed using the HC-10 antibody that detects denatured heavy chains [38], and W6/32 that binds to the combined epitope contributed by the α2 and α3 domains of the heavy chain and β2m, dependent on the presence of peptides in the peptide-binding groove [40-42]. The levels of open conformers were clearly detectable in DAOY and to a lesser degree in D556 medulloblastoma cell lines that display relatively high levels of MHC class I antigens (Fig. 3A). In contrast, open conformers were virtually non-existent in MHC class I low D283 cell line. To determine whether β2m can bind to open conformers we evaluated the effect of HC-10 antibody on β2m binding to DAOY cells (Fig. 3B). We focused on DAOY cells because they displayed the highest levels of open conformers. An increase in the levels of β2m observed following addition of exogenous β2m was largely prevented by HC-10, but not W6/32 antibody, suggesting that most of the exogenous β2m binds to open forms of HLA class I. However, residual β2m bound even in the presence of HC-10, likely reflecting its specificity. Molecular HLA class I typing of DAOY cells revealed a genotype consisting of A*0101, A*0201, B*0703, B*5701, Cw*0602 and Cw*0702 (data not shown), of which only A*0101 and A*0201 are not recognized by HC-10 [38]. Finally, we performed flow cytometry to determine whether binding of exogenous β2m modulates the ratio of open to closed cell surface conformers. As predicted, addition of exogenous β2m to DAOY cells reduced the relative levels of open, and increased the levels of closed conformers (Fig. 3B), suggesting that binding of exogenous β2m can alter the balance between the open and closed conformers.

### Engagement of open MHC class I conformers modulates phosphorylation of ERK1/2

**Figure 4:**
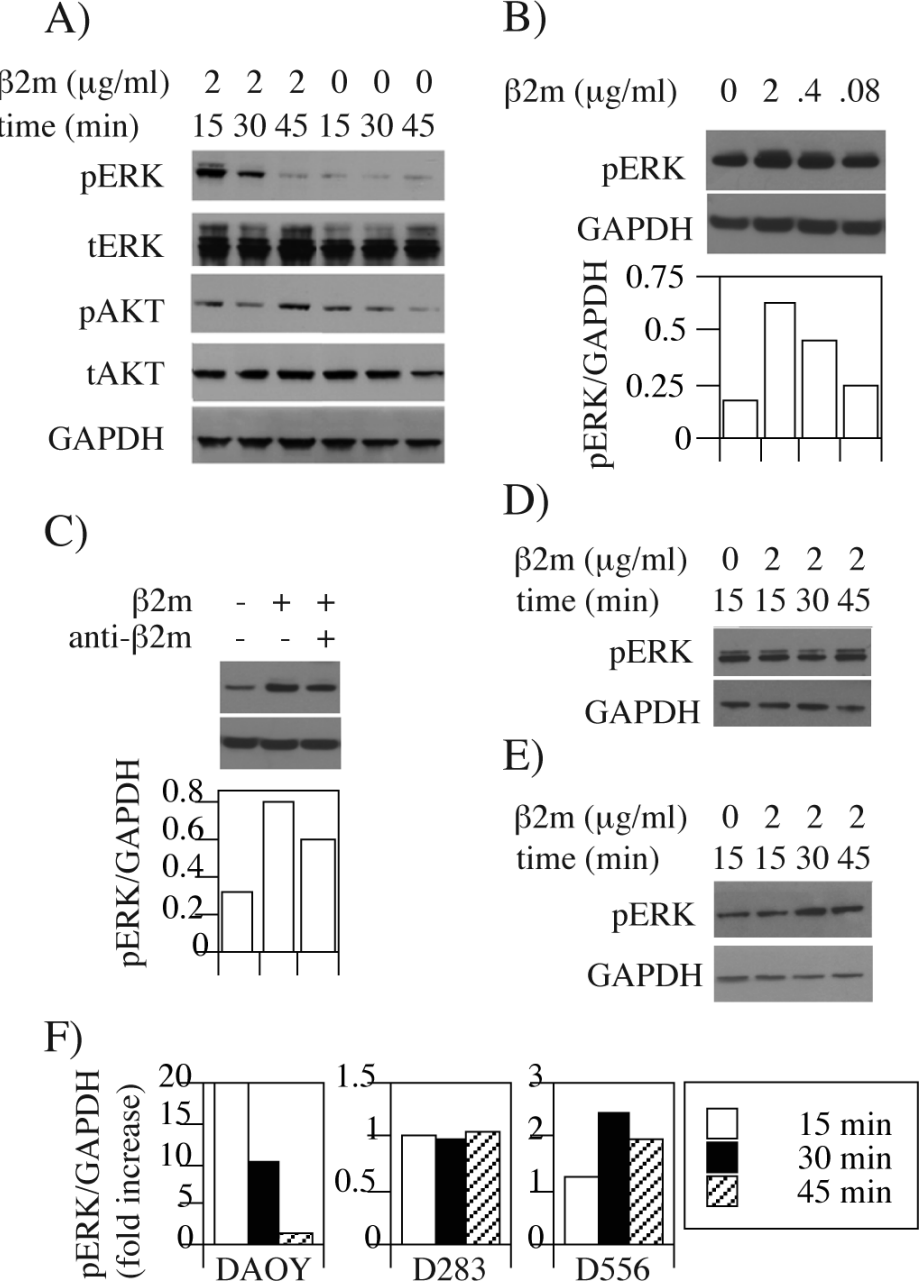
**Increased phosphorylation of ERK1/2 in DAOY and D556 cells in response to exogenous β2m**. A) Serum starved DAOY cells were incubated in the presence or absence of human β2m (2 μg/ml). At indicated times after β2m addition, cells were lysed and the lysates were analyzed by Western Blotting for phosphorylated (pERK) and total ERK, as well as for GAPDH as a control for loading. B) DAOY cells were treated with decreasing concentrations of β2m, and analyzed for phosphorylation of ERK1/2 15 minutes post β2m addition as in (A). Densitometric quantification of pERK1/2 bands relative to GAPDH in each of the treatments is shown below representing 3.65- (2 μg/ml), 2.65- (0.4 μg/ml), and 1.44- (0.08 μg/ml) fold increase in pERK1/2 over the background observed in the absence of β2m. C) β2m (2 μg/ml) was mixed with anti-β2m antibody (0.2 mg/ml) prior to the addition to DAOY cells and ERK1/2 activation was analyzed 15 minutes post β2m addition. Densitometric quantification is shown below indicates 43% inhibition by anti-β2m antibodies of β2m-induced ERK1/2 phosphorylation. D-E) The effect of β2m (2 μg/ml) incubation of indicated time lengths on phosphorylation of ERK1/2 in D556 (D) or D283 (E) cells was examined by Western blotting. F) Summary of pERK1/2 quantification in three different cell lines at indicated times after β2m treatment, relative to the levels in untreated cells (results are expressed as fold induction).

**Figure 5:**
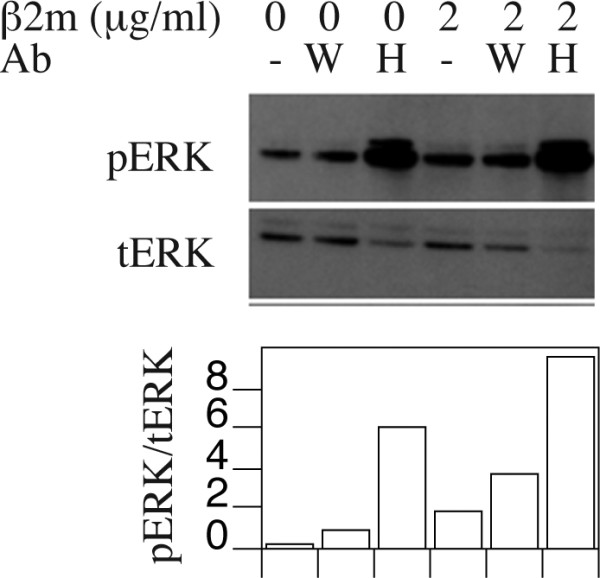
**Phosphorylation of ERK1/2 by an antibody specific for open forms of HLA class I**. Exogenous β2m in the absence or presence of W6/32 (W) or HC-10 (H) monoclonal antibodies was added to DAOY cells. Cell lysates were probed by phospho-ERK- or total ERK- specific antibodies. Quantitative ratio of phospho-ERK1/2 to total ERK1/2 is shown below.

We next examined whether altering balance between the open and closed MHC class I conformers may contribute to signal modulation. We therefore evaluated the impact of exogenous β2m on phosphorylation of ERK1/2 and AKT that are downstream arms of many receptor pathways. Increased levels of phospho-ERK1/2 were found 15–30 minutes following addition of exogenous β2m to DAOY cells (Fig. 4A), while the levels of phospho-AKT remained largely unaltered. The optimal increase in ERK1/2 phosphorylation was achieved with 2 μg/ml β2m (Fig. 4B), which is well within the range of physiological concentration in human serum [26,27,29]. Further, the effect was partially inhibited by anti-β2m antibodies despite their 10-fold molar deficit (Fig. 4C). Because there is a constitutive low level of ERK1/2 phosphorylation in the absence of any treatment, we refer to the effect of β2m as modulation, rather than induction of ERK1/2 phosphorylation. Consistent with the available levels of open conformers, increased levels of pERK1/2 were seen in D556 cells, albeit with a slightly delayed kinetics (Fig. 4D), but not in MHC class I deficient D283 cells (Fig. 4E). We next tested whether HC-10 antibody could prevent β2m -induced ERK1/2 phosphorylation. We were surprised to see that adding HC-10 itself modified ERK1/2 activation in a similar manner as β2m (Fig. 5). This was not the case with W6/32 antibody that recognizes closed HLA class I conformers. Therefore, two independent open conformer ligands enhance ERK1/2 phosphorylation in DAOY cells.

### Increased migration of DAOY cells in the presence of β2m

**Figure 6:**
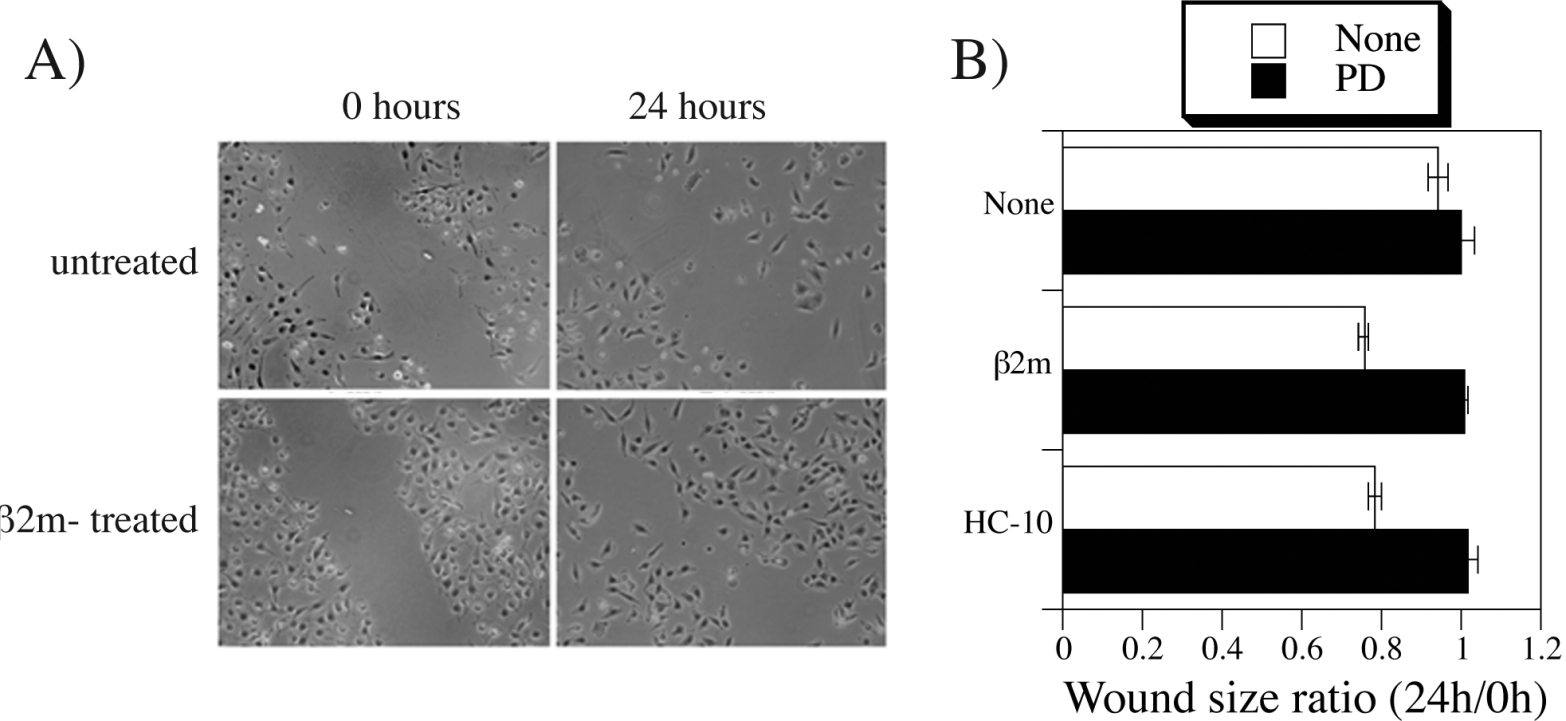
**β2m stimulates in vitro migration of DAOY cells**. A) Following introduction of a scratch wound in a near-confluent layer, DAOY cells were cultured in the absence or presence of 2 μg/ml β2m, as indicated. Shown are representative photographs of the same areas immediately after the scratch (time 0) and 24 hours later. B) Shown are mean and standard errors of the ratios of square areas of the wounds measured at 0 and 24 hour time points in triplicate cultures treated with β2m, HC-10, or media alone, in the presence or absence of 100 μm PD98059, as indicated.

Given the previously established association of higher expression of β2m with metastatic disease in medulloblastoma [35], we wondered whether engagement of open conformers might affect the migration characteristics of medulloblastoma cells in a wound scratch assay. DAOY cells were grown into near confluence when a wound was created in the center of the monolayer, and the ability of neighboring cells to migrate into the denuded area within 24 hours was determined. While control DAOY cells showed marginal ability to invade, the presence of β2m clearly induced significant migratory activity (Fig. 6A). Evaluation of the surface area of the scratch at 0 and 24 hours time points indicated that untreated cells recolonized only 6%, while the β2m- and HC-10-treated recovered 24% and 22% of the wound area, respectively (Fig. 6B). The migration was inhibited in the presence of 100 μm PD98059, pharmacologic inhibitor of upstream member of the ERK1/2 activation pathway [52]. Thus, the ERK1/2 activation enhanced by the engagement of open HLA class I conformers may contribute to higher migratory capacity of medulloblastoma cells.

## Discussion

We show herein that classical MHC class I is undetectable in the majority of medulloblastomas. HLA expression by medulloblastoma was reported twice previously. Bodey et al. analyzed the infiltrating cell phenotype in 34 medulloblastomas and 42 astrocytomas [46]. The authors noted that neoplastically transformed cells expressed HLA-A, -B, and -C molecules within all 76 tissues. Since this study focused on the phenotype of infiltrating cells little detail was provided about HLA expression by tumor cells. In another study of 10 medulloblastomas, none of the tumors showed HLA expression [47]. One major advantage of this study is that MHC class I processing machinery was analyzed in greater detail, by examining expression of HLA heavy and light chains and TAP2 subunit. In addition, other molecules, such as LMP2 and LMP7 that are involved, but not essential for MHC class I processing, were studied. The present investigation combined elements from both previous ones, including a large patient sample size and analysis of expression of several molecules required for HLA class I assembly. The large sample size enabled us to detect a minority of medulloblastomas that express HLA class I, that may be easily missed in smaller sample collections. Importantly, we were able to exclude infiltrating leukocytes as a source of HLA class I expression and analyze it in relation to the histological subtypes of medulloblastoma.

MHC class I- negative tumor cells may arise due to multiple mutations in genetically unstable cancer cells and subsequent selection by tumor-specific CD8^+ ^T cells [7,8]. The negative prognostic impact of a loss of MHC class I in small cell lung carcinoma, pancreatic carcinoma, cervical cancer, colon cancer and melanoma is consistent with this view [9-12]. Alternatively, HLA-negative may have arisen by transformation of cells that originally did not express MHC class I. These examples would include well-differentiated cells, such as muscle cells and neurons, unless the expression is induced by inflammatory stimuli. Because medulloblastoma develops from neuronal precursors and the CNS is relatively protected from the immune system, we consider selection by CD8^+ ^T cells an unlikely cause of the HLA-negative phenotype of most medulloblastomas. An explanation for HLA class I expression by a minority of medulloblastomas remains to be established.

The expression of c-myc and/or presence of anaplasia, which are negative prognostic markers for medulloblastoma [48-51], were associated with HLA class I expression. Thus, HLA class I expression may be associated with more aggressive medulloblastomas. If the association is confirmed in larger studies, MHC class I expression may prove to be an important biomarker of the malignant phenotype. Because follow-up clinical data were not available for all patients in this study, we were unable to make correlation between high HLA class I expression and disease outcomes. Nevertheless, the association of higher expression of β2m with metastatic disease in a previous study of ours [35] is consistent with the findings of the present study. Despite considerable evidence suggesting that the loss of MHC class I by tumor cells may indicate escape from immune surveillance, there are recognized examples, including non-small cell lung cancer, uveal melanoma and breast carcinoma, where increased survival correlates with downregulation of MHC class I [15-17]. This paradoxical effect may be mediated by the activity of NK cells, a cell type that is generally more effective when target cells do not display MHC class I [8]. Alternatively, β2m [23] and heavy chain devoid of peptide and β2m [19], may be implicated in modifying signal transduction, suggesting an immune system-independent role of MHC class I subunits in tumor progression. Indeed, the latter possibility was demonstrated by the ability of β2m to modulate the phosphorylation of ERK1/2. Binding of the exogenous β2m to open forms of MHC class I was likely responsible for this effect because antibody specific for the HLA open forms inhibited the binding and could mimic the signal modulation effect.

**Figure 7:**
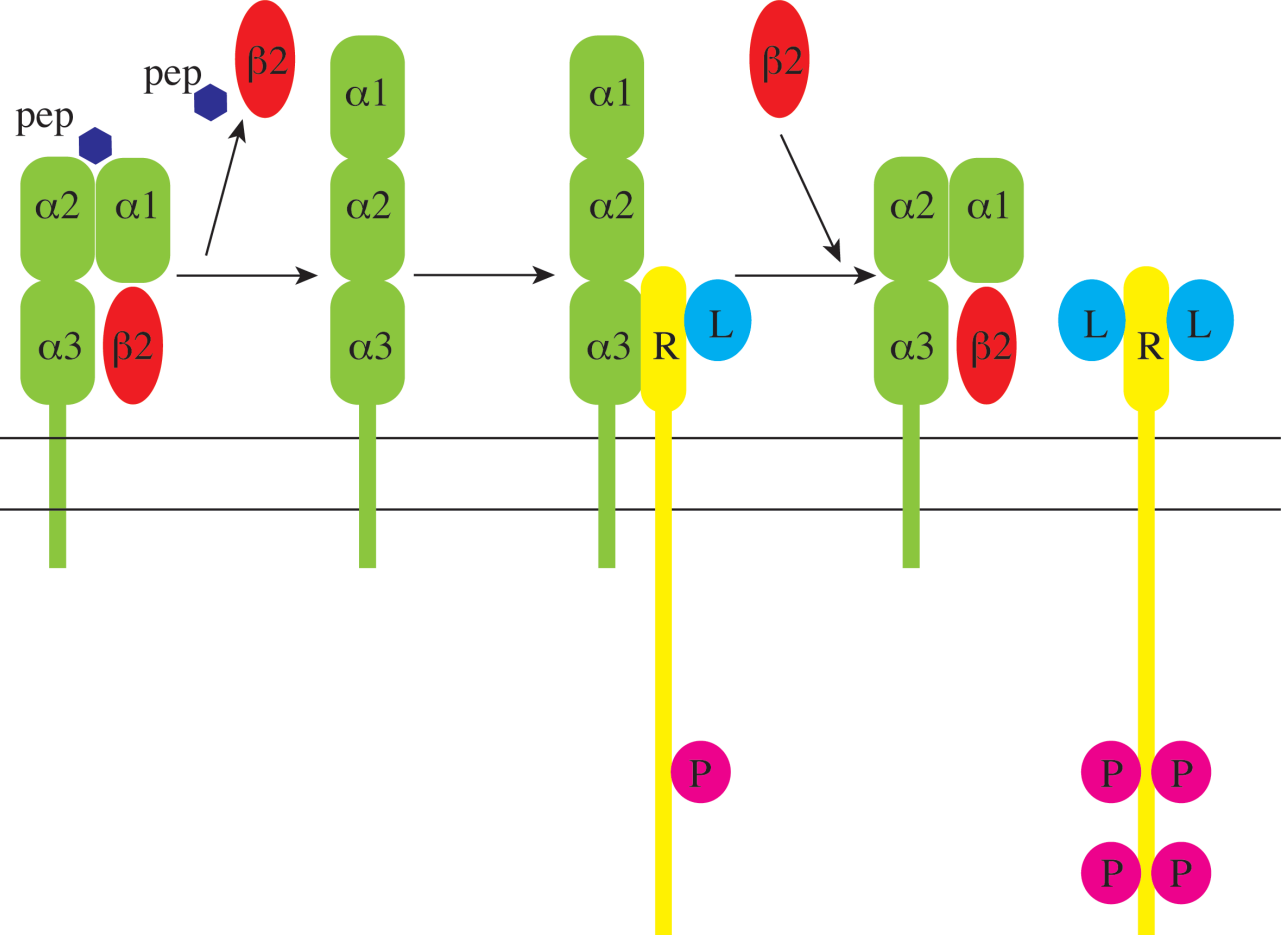
**Schematic representation of signal transduction modification by MHC class I open conformers in medulloblastoma**. The closed conformation of MHC class I (left) is composed of the heavy chain possessing three extracellular domains (α1, α2 and α3) non-covalently bound to β_2_m (β2) and antigen peptide (pep). Dissociation of β_2_m and peptide leads to the formation of open conformation that can interact with receptors (R) on the cell surface. This interaction dulls the impact of receptor ligand (L) binding (here symbolically represented by binding of one unit of ligand per one unit of receptor; note, however, that the impact of open conformers may not be related to ligand binding, but some other mechanism, i.e. preventing optimal receptor conformation). Binding of extracellular β_2_m to open conformer releases the receptor, enabling full blown signaling indicated by increased phosphorylation (P) of intracellular portion of the receptor.

Although the MHC class I molecule is not a signaling molecule on its own, the literature is replete on its involvement in signaling. Thus, cross-linking MHC class I molecules by antibodies in Jurkat T cells and T cell clones induces TCR activation similar to that induced by TCR engagement [53,54]. Neither cytoplasmic nor transmembrane domain of heavy chains are required for signal transmission, suggesting that MHC class I molecules associate and use the signal transduction machinery of other cell surface receptors [55]. Further, addition of β2m can reduce [20] or enhance [21-24] signal transduction by different receptors (Fig. 7), confirming the ability of open MHC class I conformers of modifying the function of various cell surface receptors [19]. ERK1/2 activation in medulloblastomas can occur following activation of growth factor receptors, such as EGFR, PDGFR, IGF1R and CXCR4 [56-59]. However, we found no increase in phosphorylation of these receptors following engagement of the open conformers (data not shown). Consistent with this notion is also the fact that we observed no consistent increase in phosphorylation of Akt that is normally activated by EGFR, PDGFR, IGF1R and CXCR4 receptors. Alternatively, the asymmetric signaling may result from the selective (in)action of protein phosphatase 2A (PP2A). PP2A is activated in medulloblastomas [60] and can selectively inactivate Akt or ERK1/2 due to binding of distinct regulatory subunits [61]. Thus, the exact mechanism of HLA class I open conformer engagement-mediated activation of ERK1/2 in medulloblastoma remains to be determined.

Dysregulation of ERK1/2 has been implicated in tumorigenesis of various cell types. Mitogen-activated protein kinases are involved in the cellular response to stimuli resulting in activation of membrane, cytoplasmic and nuclear signaling pathways. ERK1/2 has a role in the phosphorylation of cytoplasmic and nuclear targets, regulation of cell proliferation, differentiation, survival, angiogenesis, migration and chromatin remodeling [62,63]. Our results show a role of ERK1/2 activation in promoting migration of medulloblastoma cells in vitro. This may explain previously found higher levels of β2m expression in metastatic medulloblastoma [35]. Excess of β2m protein produced and secreted into the extracellular matrix of medulloblastoma tissues could bind to HLA class I open conformers on neighboring cells, enhancing activation of ERK1/2 and the invasive capability of medulloblastoma cells.

In summary, we found a novel role of MHC class I contributing towards a more malignant phenotype of medulloblastoma by enhancing the activation of ERK1/2. The contrasting effects of MHC class I on different tumors could be explained by differential effects of MHC class I on individual cell surface receptor signaling and/or by the presence or absence of an overriding tumor-specific CD8+ T cell response.

## Abbreviations

β2m: β2-microglobulin; IHC: immunohistochemistry; TAP: transporter associated with antigen processing.

## Competing interests

The authors declare that they have no competing interests.

## Authors' contributions

CS carried out IHC staining (except for c-myc), immunofluorescence staining, PCR, Western blot and migration experiments, analyzed results and participated in the writing of the manuscript. MS participated in the generation of the tissue array and performed analysis of IHC. BR performed flow cytometry analysis. EJR performed analysis of IHC and participated in the writing of the manuscript. MRC performed staining and analysis for c-myc expression. BRR participated in the study design, recruited medulloblastoma patients and isolated RNA from medulloblastoma specimens. RC designed and constructed the custom tissue microarray. TJM participated in the study design, tissue microarray generation and writing of the manuscript. SV designed the study, analyzed and interpreted the data, and participated in all phases of manuscript writing. All authors have read and approved the final manuscript.
